# Forecasting provincial agricultural output value in China via multiple nighttime light indices and neural networks

**DOI:** 10.1038/s41598-025-22808-3

**Published:** 2025-11-10

**Authors:** Rongchao Yang, Qingbo Zhou, Lei Xu, Zijuan Zhao, Yi Zhang, Tongyang Wei

**Affiliations:** 1 Institute of Agricultural Information, Chinese Academy of Agricultural Sciences, Beijing, 100081 China; 2 Institute of Agricultural Resources and Regional Planning, Chinese Academy of Agricultural Sciences, Beijing, 100081 China

**Keywords:** Nighttime light (NTL) remote sensing data, The total output value of agriculture, Forestry, Animal husbandry, Fishery (TOVAFAF), Forecasting model, Neural networks, Ensemble optimization mechanism, Sustainability, Information technology, Electrical and electronic engineering

## Abstract

It is crucial for comprehending the developmental trend of the agricultural economy, refining the agricultural industrial structure, and amending agricultural policies to accurately forecast and timely obtain the total output value of agriculture, forestry, animal husbandry, and fishery (TOVAFAF). This paper attempts to utilize eight nighttime light (NTL) indices originally constructed based on NPP-VIIRS NTL remote sensing data serving as multiple input variables to establish a more accurate and effective forecasting model for the TOVAFAF in various provinces of China. For the major challenge of characterizing the complex nonlinear relationship between NTL data and the TOVAFAF under the condition of limited samples, this paper employed single-hidden-layer back propagation (BP) neural network and extreme learning machine (ELM) as two basic modeling methods, proposed a novel ensemble particle swarm optimization (EPSO) algorithm as optimization mechanism for neural network models to overcome the limitation of traditional particle swarm optimization (PSO) algorithm, and used logarithmic transformation to enhance the correlation between NTL data and the TOVAFAF. The experimental results further substantiate that the neural network algorithms can effectively characterize the potential nonlinear relationship between NTL data and the TOVAFAF. And the neural network models optimized by the EPSO mechanism, those under logarithmic transformation, and the BP neural network series models exhibit superior forecasting performance than those optimized by the PSO mechanism, those under normalization, and the ELM series models, respectively. Furthermore, the EPSO-BP model under logarithmic transformation provides the best forecasting performance on the TOVAFAF for China’s various provinces in 2023, with the mean relative error (MRE) of 20.65% and the determination coefficient (R^2^) of 0.8749 for the linear relationship between the actual and forecasting values, which presents a decrease of 11.55 percentage points in MRE and an increase of 35.38% in R^2^ compared to the PSO-ELM model in our previous research.

## Introduction

Agriculture is the pillar industry of China’s national economic development, playing a fundamental and supportive role in the operation of the entire economic system, social stability, and coordinated development^1,2^. As the fundamental industry of the three major industries, the development level of agriculture is closely related to the people’s quality of life and the long-term stability of society^1^. With the continuous adjustment and optimization of agricultural policies, China’s agriculture has changed from the traditional agriculture dominated by planting industry to the modern agriculture with the comprehensive development of agriculture, forestry, animal husbandry and fishery, and the comprehensive agricultural production capacity has steadily increased^3^. The total output value of agriculture, forestry, animal husbandry, and fishery (TOVAFAF) can reflect the overall achievements and scale of the comprehensive agricultural production during a certain period^3,4^. Therefore, accurately forecasting and obtaining the TOVAFAF plays an important role in grasping the development trend of agricultural economy, optimizing agricultural industrial structure, and adjusting policies^3^.

At present, the data and indicators used to measure the level of socio-economic development mainly come from economic census or surveys organized by National Bureau of Statistics or other administrative departments^5,6^. But this method requires a significant investment of manpower, financial resources, and time^7^. Moreover, it is difficult to obtain socio-economic data in some remote areas, and the survey efficiency is low^8^. Therefore, it is necessary to establish low-cost, timely, and accurate methods to obtain socio-economic data. Some scholars employed socio-economic time-series data to establish forecasting models, such as grey model^9^, autoregressive integral moving average (ARIMA) model^10^, and combination forecasting model^11,12^, which provided technical support for timely and accurate acquisition of socio-economic data and indicators. However, such time-series forecasting models have a temporal dependence on the socio-economic time-series data points, so they need to rely on traditional survey methods to obtain socio-economic data for a certain time point or time period as training data to a certain extent, so as to make more accurate prediction of socio-economic data for the subsequent time point or time period.

Benefiting from the development of remote sensing technology, nighttime light (NTL) remote sensing technology possesses a unique ability to detect and record low-level visible and near-infrared electromagnetic spectrum information emitted from the Earth’s surface, such as the lights of residential areas, buildings, streets, traffic, and so on^13–15^. The NTL radiation intensity can generally reflect the intensity of human activities and the level of socio-economic development^16^. Elvidge et al.^13^ have demonstrated that the area lit by anthropogenic visible-near infrared emissions was highly correlated with gross domestic product (GDP) and electric power consumption, which provided a theoretical basis for the application of NTL data to the quantification of human activities and the assessment of socio-economic development level. Moreover, NTL remote sensing images have the advantages of wide coverage, space–time continuity, short periodicity, availability, independence and objectivity^17,18^. Therefore, NTL remote sensing images have been widely used in the socio-economic field in recent years, such as the prediction and assessment of GDP^5,19^, electricity consumption^20,21^, population^22,23^, poverty level^24,25^, and urbanization process^26,27^.

In related studies, some scholars utilized indices constructed from NTL data to directly characterize the level and differentiation of socio-economic development^28–30^. For example, Hua et al.^29^ employed the county-level NTL indices constructed from NTL data as direct proxy variables to evaluate the poverty reduction effects of 831 national level poverty-stricken counties and 14 concentrated contiguous poverty-stricken areas in China. Moreover, some scholars utilized NTL indices modified by socio-economic, geographical, or other relevant data to characterize the level and differentiation of socio-economic development^15,31^. For example, Zhong et al.^15^ employed the weighted Theil index constructed by NTL data, GDP, and population to measure and analyze the development gap of Jiangxi Province from 2013 to 2018. In fact, these studies took NTL indices as the proxy variables for socioeconomic parameters. Although NTL data correlate strongly with socio-economic parameters, they are not perfectly correlated, which may lead to volatile and uncertain results. To a certain extent, these studies can only qualitatively characterize the level and differentiation of socio-economic development. Furthermore, some scholars used NTL indices to invert and estimate socio-economic data or indicators by constructing regression models^32–34^, so as to quantitatively evaluate the level and differentiation of socio-economic development. However, most of these studies took a single NTL index as the independent variable to construct traditional regression models, such as linear, exponential, and logarithmic models. That may not be a good reflection of the potential relationship between night light data and socio-economic data in some cases. For example, Han et al.^35^ established a log-linear regression model for the output value of primary industry based on a single NTL area index, with the determination coefficient for only 0.306. To address this issue, Yong et al.^25^ utilized back propagation (BP) neural network optimized by particle swarm optimization (PSO) algorithm (PSO-BP) and multiple NTL indices to construct a fitting model for evaluating the poverty level in southwestern China, and achieved ideal results.

TOVAFAF is considered as an important indicator to evaluate the level of agricultural economy development. This paper aims to establish an accurate and timely forecasting model for the TOVAFAF. Due to the fact that NTL data mainly comes from urbanized areas, traditional models established based on single NTL index cannot well reflect the potential relationship between NTL data and the TOVAFAF. Inspired by the reference^25^, eight NTL indices were constructed based on NTL data and statistics in our previous research, and selected four NTL indices that were significantly correlated with the TOVAFAF to establish linear and nonlinear forecasting models for the TOVAFAF in various provinces of China based on single NTL index and multiple NTL indices, so as to explore the potential relationship between NTL data and the TOVAFAF^36^. The results of our previous research^36^ indicate that the relationship between NTL data and the TOVAFAF is not a simple linear relationship, but a more complex nonlinear relationship. And the extreme learning machine (ELM) optimized by particle swarm optimization (PSO) model, called PSO-ELM model, established based on multiple NTL indices can better characterize the potential nonlinear relationship between NTL data and the TOVAFAF than other traditional regression models established based on single NTL index^36^. However, the mean relative error (MRE) of the PSO-ELM model for forecasting the TOVAFAF in various provinces of China is 32.20%, indicating that the forecasting accuracy for TOVAFAF still has great room for improvement.

The major challenge for this study is how to more effectively characterize the complex nonlinear relationship between NTL data and the TOVAFAF under the condition of limited samples. Deep learning algorithms possess strong nonlinear fitting capabilities, but they contain a large number of parameters and require a large number of samples for training and fitting. However, the limited samples in this study cannot effectively train deep learning algorithms. As a type of single-hidden-layer neural network, ELM has been demonstrated by our previous research^36^ to have the potentiality to characterize the nonlinear relationship between NTL data and the TOVAFAF under the condition of limited samples. In addition, single-hidden-layer BP neural network typically has the ability to learn and simulate nonlinear relationship in the case of limited samples. Therefore, this paper focuses on employing single-hidden-layer BP neural network and ELM as two basic neural network algorithms, and improves them to further explore the nonlinear relationship between multiple NTL indices and the TOVAFAF, aiming at improving the forecasting accuracy for the TOVAFAF.

The main innovations and contributions of this paper are: (1) to propose a novel ensemble particle swarm optimization (EPSO) algorithm as optimization mechanism for neural network models, which significantly improves the forecasting performance of neural network models for the TOVAFAF under the circumstance of limited samples; (2) to analyze and discuss the forecasting performance of different neural network models for the TOVAFAF under different optimization mechanisms and different NTL data transformations; (3) to propose a more effective framework for forecasting the TOVAFAF in China’s various provincial-level administrative regions based on multiple NTL indices.

## Materials and methods

### Study area and data sources

**Fig. 1:**
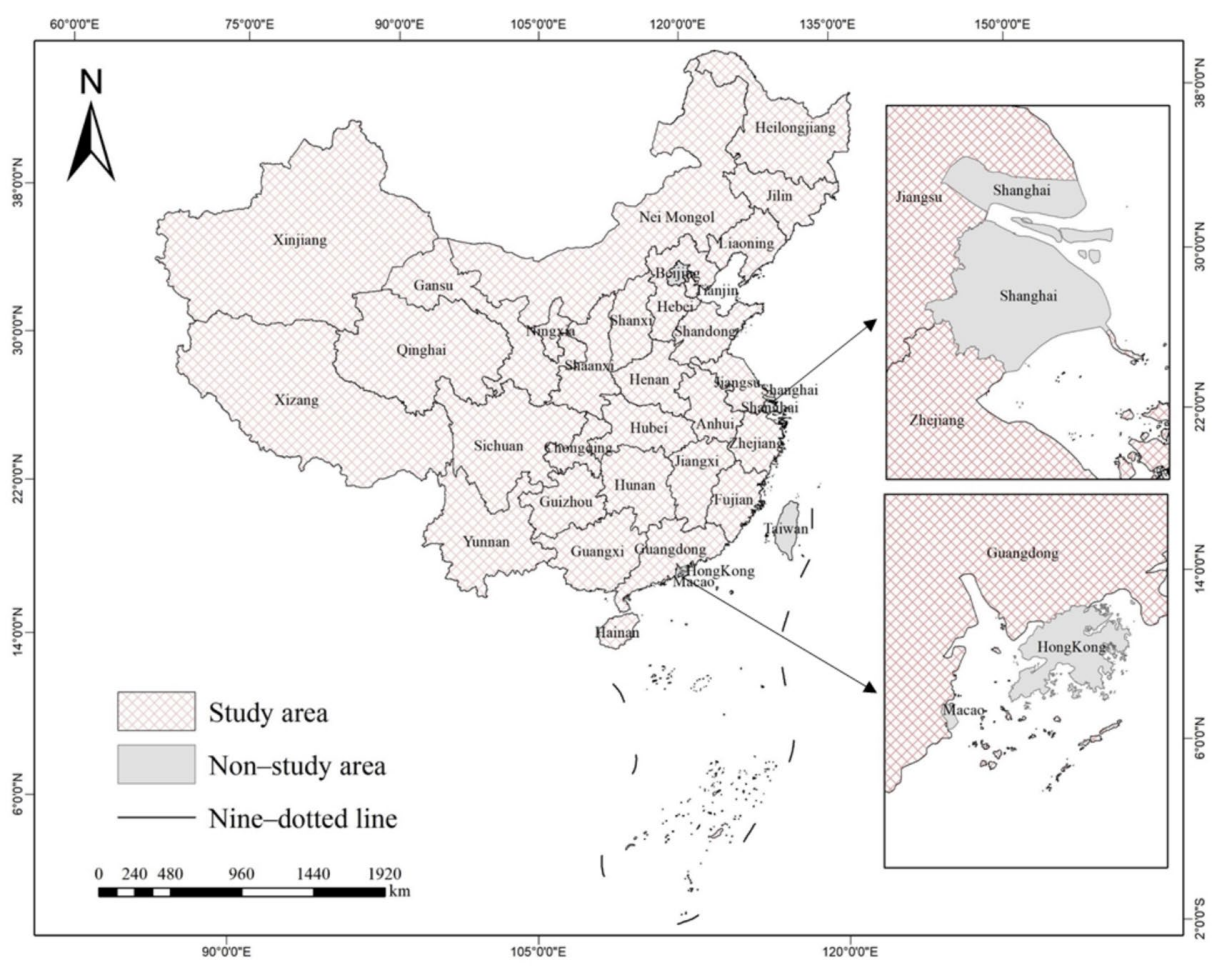
The various provincial-level administrative regions in China.

The scope of this research encompasses a range of provincial-level administrative regions across China. It is important to note that the study area of this paper does not include Beijing, Shanghai, Taiwan, Macao, and Hong Kong of China. Beijing and Shanghai concentrate on the advancement of the tertiary industry, whereas the contribution of the primary industry (encompassing agriculture, forestry, animal husbandry, and fishery) to the total economic output is relatively minor and has exhibited a decreasing trend in recent years, setting them apart from other provinces in China. The exclusion of Taiwan, Macao, and Hong Kong of China from the study area is due to insufficient relevant economic statistical data. Consequently, the remaining 29 provincial-level administrative regions are selected as the focus of this research. Figure 1 shows the various provincial-level administrative regions in China.

The National Oceanic and Atmospheric Administration (NOAA) of the United States has provided two of the most frequently utilized NTL data sources^25,37^. One of the data sources is obtained from the Defense Meteorological Satellite Program-Operational Linescan System (DMSP-OLS), which offers a global NTL map spanning from 1992 to 2013 with a spatial resolution of 30 arc-seconds, but has some shortcomings, such as coarse spatial resolution, saturation on bright lights and limited dynamic range^38^. The other is obtained from the Suomi National Polar-orbiting Partnership-Visible Infrared Imaging Radiometer Suite (NPP-VIIRS), which provides a more recent global NTL map starting from 2012 to the present, boasting a spatial resolution of 15 arc-seconds. The NPP-VIIRS dataset stands out for its superior spatial resolution and its immunity to the issue of brightness saturation. Moreover, it is regularly updated, making it an ideal choice for ongoing research. Consequently, the NPP-VIIRS dataset is taken as the NTL data source of this research. To ensure the quality of NTL images, this research has selected the annual VIIRS nighttime lights version 2 (VNL V2) product from the NPP satellite, derived from monthly cloud-free average radiance grids^39^. This dataset is available for download at https://eogdata.mines.edu/products/vnl/.

Furthermore, the boundary data for China’s various provincial-level administrative regions has been acquired from the Resource and Environmental Science Data Registration and Publishing System of Chinese Academy of Sciences (website: https://www.resdc.cn/Default.aspx). As for the data on TOVAFAF within China’s various provincial-level administrative regions, it has been sourced from the website of the National Bureau of Statistics of China (website: https://data.stats.gov.cn/index.htm). It should be noted that the temporal coverage of the NPP-VIIRS NTL images and the TOVAFAF for China’s various provincial-level administrative regions spans from 2013 to 2023 in this research. Both the NPP-VIIRS NTL data and the TOVAFAF statistics used in this paper are annual (yearly) datasets, so the forecasting period of model is yearly.

### Data preprocessing

NTL remote sensing technology serves to detect the intensity of artificial lights at night, thereby characterizing the level of socio-economic development, in which the digital number (DN) value is used to represent the light intensity for each pixel of the NTL images. The annual VNL V2 product derived from NPP-VIIRS NTL data has been preprocessed to remove sunlight, moonlight, and cloudy pixels from the NTL images. And the twelve-month median radiance has been utilized to eliminate high and low radiance outliers, effectively filtering out the majority of fires and isolating the background. Furthermore, the DN values of background areas were set to zero using the data range threshold derived from 3 × 3 grid cells. The above preprocessing steps are crucial for minimizing adverse impacts of non-artificial lights on the measurement of artificial NTL intensity.

However, the DN values of some pixels still are negative values or abnormal maxima in the annual VNL V2 product. The negative values may be caused by noise generated by the sensor during the data acquisition process, while the abnormal maxima may be attributed to biomass burning or other environmental factors that have significantly impacted the data. Thus, referring to the literature^40^, the NTL images from the annual VNL V2 product were subjected to additional preprocessing using ArcGIS 10.2 software. The specific preprocessing steps are: (1) to extract the NTL images for various provincial-level administrative regions of China from 2013 to 2023 using the boundary data; (2) to convert the GCS_WGS_1984 geographic coordinate system of the NTL images into the Asia_Lambert_Conformal_Conic projection coordinate system; (3) to resample and define the spatial resolution of the NTL images as 500 m * 500 m; (4) to define negative DN values in the NTL images as zero to remove noise; (5) to take the maximum DN value in the annual NTL images of Beijing, Shanghai, Guangzhou, and Shenzhen four first-tier cities in China as the highest radiance value in the country for that year to remove the abnormal maxima.

**Fig. 2:**
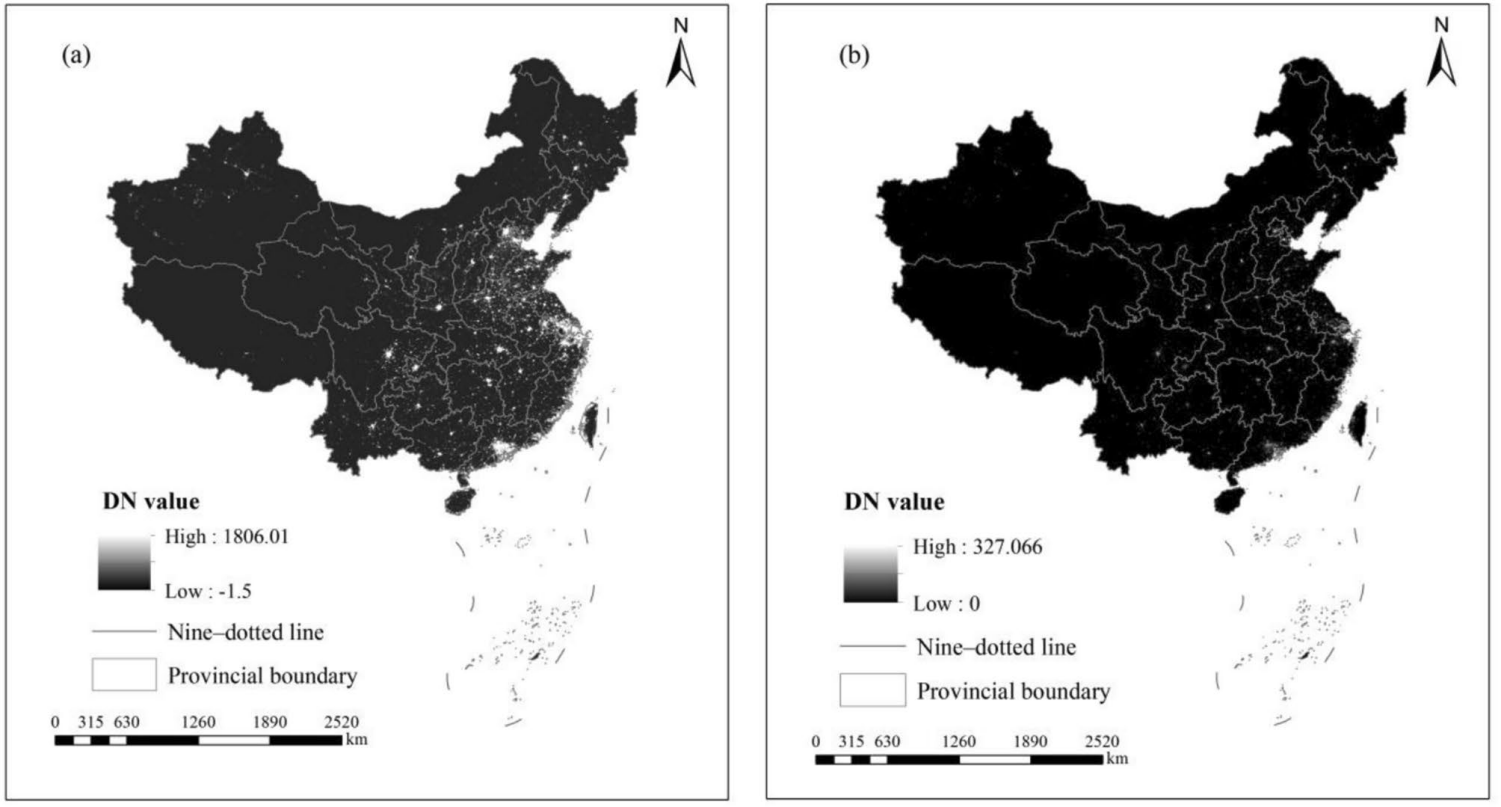
The NPP-VIIRS NTL images of China’s various provincial-level administrative regions in 2021: (**a**) the NTL image before preprocessing and (**b**) the NTL image after preprocessing.

It should be clarified that the “maximum DN value” mentioned in Step (5) refers to the maximum value among all the pixels within the four first-tier cities in the given year. The primary rationale is that the four first-tier cities represent China’s top urbanization scale and economic density. Their NTL brightness (DN value) is closely related to human activities, naturally ranking among the highest levels in China. If a non-first-tier province exhibit a single-pixel DN value exceeding the maximum recorded in Beijing, Shanghai, Guangzhou, or Shenzhen, such data is highly likely to be anomalous (not caused by human activities). Figure 2 illustrates the NTL images of China’s various provincial-level administrative regions before and after preprocessing in 2021. It can be seen that the brightness of the preprocessed NTL image in Fig. 2(b) is darker than that of the unprocessed NTL image in Fig. 2(a), which is the result of removing abnormally high values caused by biomass combustion or other environmental factors. Although visually “darkening”, the processed data better conforms to the distribution pattern of NTL on the surface caused by human economic activities, providing a more reliable input for subsequent modeling.

### Construction for NTL indices

Inspired by the reference^25^, eight NTL indices were constructed using various statistical measures in our previous research^36^. However, only four NTL indices that were significantly correlated with the TOVAFAF were selected to establish the forecasting model for the TOVAFAF in China’s various provincial-level administrative regions. This paper attempts to strengthen the correlation between the TOVAFAF and the NTL indices by logarithmic transformation on the basis of the eight NTL indices originally constructed, so as to make full use of the eight NTL indices to establish a more accurate TOVAFAF forecasting model. Table 1 lists and describes the eight NTL indices constructed in our previous research^36^.

**Table 1:** Detail description for the constructed NTL indices.

Serial number	Index name	Abbreviation	Detail description
1	Total nighttime light index	TNLI	The sum of light intensity for all pixels in the NTL image within the provincial-level administrative region
2	Average nighttime light index	ANLI	The ratio of TNLI to quantity of all pixels in the NTL image within the provincial-level administrative region
3	Luminous pixel quantity index	LPQI	The quantity of luminous pixels (brightness value greater than 0) in the NTL image within the provincial-level administrative region
4	Luminous pixel ratio index	LPRI	The ratio of LPQI to quantity of all pixels in the NTL image within the provincial-level administrative region
5	Average luminous pixel light index	ALPLI	The ratio of TNLI to quantity of luminous pixels in the NTL image within the provincial-level administrative region
6	Nighttime light standard deviation index	NLSDI	The standard deviation of light intensity for all pixels in the NTL image within the provincial-level administrative region
7	Nighttime light variance index	NLVI	The variance of light intensity for all pixels in the NTL image within the provincial-level administrative region
8	Nighttime light squared deviation sum index	NLSDSI	The sum of squared deviation of light intensity for all pixels in the NTL image within the provincial-level administrative region

### Modeling methods

Our previous research has preliminarily clarified that there is a potential nonlinear relationship between NTL data and the TOVAFAF^36^. Furthermore, the extreme learning machine (ELM) optimized by particle swarm optimization (PSO) model (referred to as PSO-ELM model) established on the basis of multiple NTL indices demonstrates a superior ability to characterize the potential nonlinear relationship, compared with the traditional regression models established based on single NTL index^36^. Based on our previous research, this paper primarily focuses on applying back propagation (BP) neural network and ELM along with their respective improved algorithms to establish the forecasting models. It should be noted that the construction of all models in this paper was completed using MATLAB R2022a software.

BP neural network is a multilayer feedforward neural network, consisting of an input layer, one or more hidden layer, and an output layer. This network continuously adjusts the network weights and thresholds through error back propagation algorithm during training to minimize the error between the forecasting output and the expected output^41^, which possess the ability to learn and simulate complex nonlinear relationship. It must be emphasized this paper utilizes single-hidden-layer BP neural network as the modeling method.

ELM is a single-hidden-layer feedforward neural network, comprising an input layer, a hidden layer, and an output layer. This network employs a random initialization for input weights and hidden layer biases, and utilizes the generalized inverse matrix theory to compute the output weights, thereby aligning the forecasting output with the expected output^42^, which eliminates the need for iterative weight adjustments during the training phase, and possesses good generalization performance and fast learning ability.

PSO is an intelligent optimization algorithm that simulates the foraging behavior of birds, exhibiting remarkable performance in solving complex optimization problems^43^. PSO initializes a batch of particles equipped with position vector ${\mathbf{X}}_{i} = {\text{[X}}_{i1} , {\text{X}}_{i2} , \, \cdots ,{\text{X}}_{iD} {]}$ and velocity vector ${\mathbf{V}}_{i} = {\text{[V}}_{i1} {,} {\text{V}}_{i2} {, } \cdots ,{\text{V}}_{iD} {]}$ in *D*-dimensional space, in which $i \in {\text{\{ 1, }}{2, } \cdots {, }N{\text{\} }}$, and *N* represents the population size of particles. These particles explore for the optimal solution in the *D*-dimensional search space through moving, interacting with each other, and perpetually iterating, in which the position and velocity in each dimension are restricted within a certain range to prevent the blind searching of particles, i.e.,${\text{X}}_{id} \in {\text{[ - X}}_{{{\text{max}}}} {\text{, X}}_{{{\text{max}}}} {]}$ and ${\text{V}}_{id} \in {\text{[ - V}}_{{{\text{max}}}} {\text{, V}}_{{{\text{max}}}} {]}$. In PSO, the position of each particle corresponds to a potential solution to the optimization problem, while its velocity dictates the direction and speed of its movement, facilitating the update of its position. The fitness function assesses the performance of each particle’s position. PSO employs the following equations to update the velocity and position of each particle:

1 $$V_{id}^{k + 1} = wV_{id}^{k} + c_{1} r_{1} (pbest_{id}^{k} - X_{id}^{k} ) + c_{2} r_{2} (gbest_{id}^{k} - X_{id}^{k} )$$

2 $${\text{X}}_{id}^{k + 1} = {\text{X}}_{id}^{k} + {\text{V}}_{id}^{k + 1}$$

where ${\text{V}}_{id}^{k}$ and ${\text{X}}_{id}^{k}$ represent the velocity and position of *i*th particle in *d*th dimension at the *k*th iteration, respectively, in which $d \in {\text{\{ 1, }}{2, } \cdots {, }D{\text{\} }}$, $k \in {\text{\{ 1, }}{2, } \cdots {, }M{\text{\} }}$, and *M* represents the number of iterations; *w* represents the inertia weight; *c*_1_ and *c*_2_ are the acceleration constants; *r*_1_ and *r*_2_ are random values distributed within [0,1];${\text{pbest}}_{id}^{k}$ represents the optimal position of the *d*th dimension for *i*th particle at the *k*th iteration; and ${\text{gbest}}_{id}^{k}$ represents the optimal position of *d*th dimension across the entire particle swarm.

This paper utilized the PSO algorithm to optimize the initial weights of BP neural network and ELM, thereby establishing PSO-BP and PSO-ELM models. Given that the initialized weights of BP and ELM vary each time, the weights optimized by PSO also differ accordingly, which may produce a certain impact on the results. To address this issue, this paper constructed an ensemble PSO algorithm as optimization mechanism for neural network models, thereby establishing EPSO-BP and EPSO-ELM models, in which PSO-BP and PSO-ELM were separately taken as the base evaluator, and the mean value of the forecasting outcomes from multiple PSO-BP and PSO-ELM models was adopted as the final result, respectively.

### Accuracy assessment

To comprehensively evaluate the forecasting performance of the constructed models, the determination coefficient (R^2^), relative error (RE), and mean relative error (MRE) were taken as evaluation metrics in this paper. R^2^ is utilized to assess the goodness of fit for the forecasting model, in which a larger R^2^ value indicates a better fitting effect of the model and a stronger explanatory power for the dependent variables. RE is employed to reflect the degree of deviation between the model’s forecasting value and the actual value for a given sample, in which a smaller RE value signifies a better forecasting performance for the sample. MRE is used to gauge the reliability of the forecasting model, in which a smaller MRE value suggests higher reliability of the model. The mathematical expressions for R^2^, RE, and MRE are as follows:

3 $$R^{2} = \frac{{\sum\limits_{i = 1}^{N} {(\overset{\lower0.5em\hbox{$\smash{\scriptscriptstyle\frown}$}}{t}_{i} - \overline{t})^{2} } }}{{\sum\limits_{i = 1}^{N} {(\overset{\lower0.5em\hbox{$\smash{\scriptscriptstyle\frown}$}}{t}_{i} - \overline{t})^{2} } }},(0 \le R^{2} \le 1)$$

4 $${\text{RE}} = \left| {\frac{{\hat{t}_{i} - t_{i} }}{{t_{i} }}} \right| \times 100\%$$

5 $$MRE = \frac{{\sum\limits_{i = 1}^{N} {\left| {(\overset{\lower0.5em\hbox{$\smash{\scriptscriptstyle\frown}$}}{t}_{i} - t_{i} )/t_{i} } \right|} }}{N} \times 100\%$$

where $t_{i}$ is the actual value of the *i*th sample, $\hat{t}_{i}$ is the forecasting value of the *i*th sample, $\overline{t}$ represents the mean of the actual values across all samples, and *N* denotes the number of all the samples.

### Sample division

This paper takes the NTL data of a certain province in a specific year and its corresponding TOVAFAF as a sample, resulting in the sample with a yearly temporal resolution. This paper collected a total of 319 samples from 2013 to 2023 covering 29 provinces in China, which were divided into training set, validation set, and test set. Specifically, the 232 samples spanning from 2013 to 2020 covering 29 provinces in China were utilized as the training set for the training and establishment of forecasting models. The 58 samples from 2021 to 2022 covering 29 provinces were designated as the validation set, serving the dual purpose of optimizing the models’ weight parameters and conducting a preliminary assessment of the modeling efficacy. The 29 samples from 2023 covering 29 provinces was designated as the independent test set, offering a rigorous evaluation of the forecasting capabilities of the established models.

## Results and discussion

In this section, BP, ELM, PSO-BP, PSO-ELM, EPSO-BP, and EPSO-ELM algorithms were employed to establish forecasting models of TOVAFAF for China’s various provincial-level administrative regions, taking the eight NTL indices (TNLI, ANLI, LPQI, LPRI, ALPLI, NLSDI, NLVI, and NLSDSI) processed by normalization and logarithmic transformation as multiple input variables, respectively. It should be noted that normalization refers to normalizing each original NTL index to the range of 0–1, in order to avoid the impact of different dimensions on the modeling efficacy. Logarithmic transformation refers to performing logarithmic operations on each original NTL index with a base of 10. The experimental results were discussed and analyzed to further explore the potential nonlinear relationship between NTL data and the TOVAFAF, so as to enhance the forecasting accuracy for the TOVAFAF.

### Parameter settings

Before modeling, it is necessary to set and optimize the parameters of various models to achieve optimal performance. This paper employed the *newff* function in MATLAB software to construct the BP model. The parameters that need to be set for BP include the number of hidden layers, number of hidden layer neurons, type of activation function, type of training function, number of training iterations, learning rate and target error, in which the setting values of these parameters are the same as the setting values of BP in PSO-BP and EPSO-BP. ELM is a single hidden layer feedforward neural network, where only two parameters need to be set, i.e., the number of hidden layer neurons and the type of activation function, in which the setting values of these parameters are the same as the setting values of ELM in PSO-ELM and EPSO-ELM. For PSO-BP, PSO-ELM, EPSO-BP and EPSO-ELM models, the parameters for PSO in the Eq. (1) need to be set. Since EPSO-BP and EPSO-ELM are ensemble models, the number of base estimators need to be set. The specific parameter settings for various models are shown in Table 2.

**Table 2:** Parameter settings for various models.

Model	Parameter	Normalization	Logarithmic transformation
BP BP in PSO-BP BP in EPSO-BP	Number of hidden layer neurons	20	19
	Iterations	100	
	Learning rate	0.01	
	Target error	0.001	
	Activation function	*tansig* for hidden layer, *purelin* for output layer	
	Training function	*trainlm*	
ELM ELM in PSO-ELM ELM in EPSO-ELM	Number of hidden layer neurons	19	35
	Activation function	*Sigmod* for hidden layer	
PSO in PSO-BP, PSO-ELM, EPSO-BP, and EPSO-ELM	Iterations	200	
	Population size	10	
	Inertia weight	*w* = 0.5	
	Acceleration constant	*c*_1_ = *c*_2_ = 1.49445	
	Velocity range of particles	${\text{V}}_{id} \in {[ - 1, 1]}$	
PSO in PSO-BP and EPSO-BP	Position range of particles	${\text{X}}_{id} \in {[ - 3, 3]}$	
PSO in PSO-ELM and EPSO-ELM	Position range of particles	${\text{X}}_{id} \in {[ - 1, 1]}$	
EPSO-BP and EPSO-ELM	Number of base evaluators	10	

### Evaluation and analysis of modeling efficacy

**Fig. 3:**
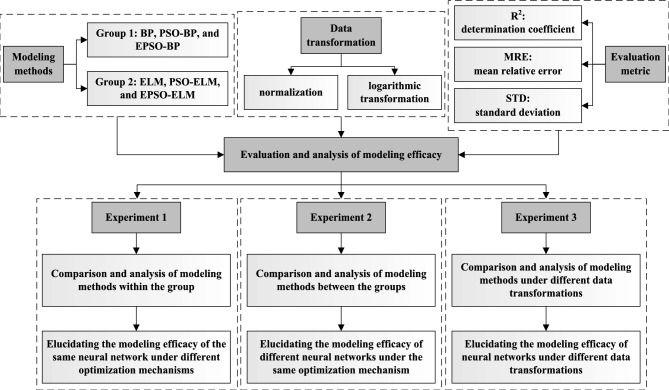
Flowchart of experimental analysis.

**Fig. 4:**
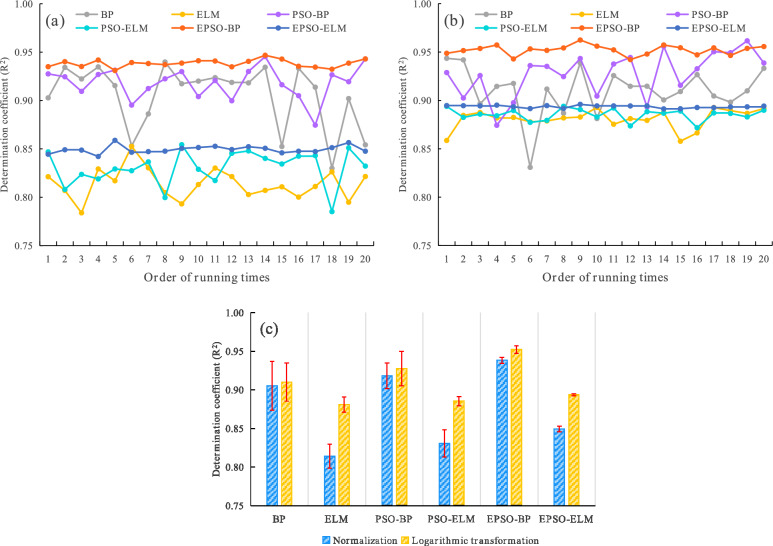
Goodness of fit for various models on the training set: (**a**) the R^2^ for various models at each run under normalization; (**b**) the R^2^ for various models at each run under logarithmic transformation; (**c**) the mean and STD of the R^2^ for various models within 20 runs under normalization and logarithmic transformation, respectively.

**Fig. 5:**
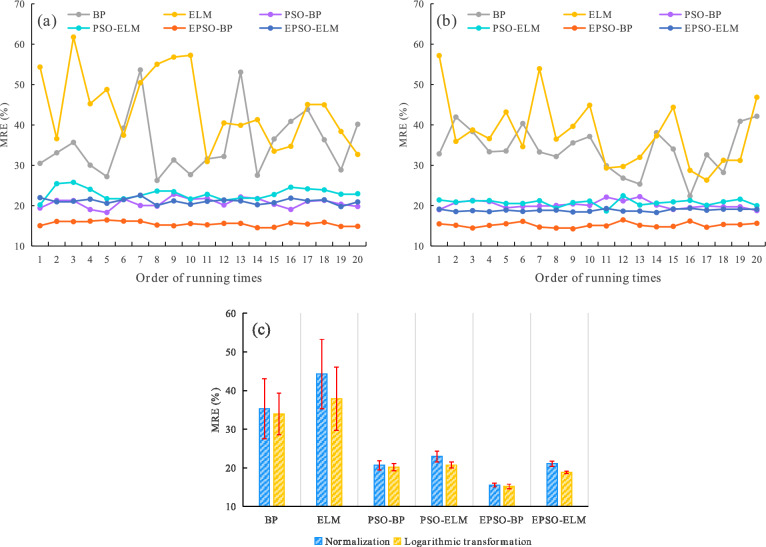
Forecasting efficacy for various models on the validation set: (**a**) the MRE for various models at each run under normalization; (**b**) the MRE for various models at each run under logarithmic transformation; (**c**) the mean and STD of the MRE for various models within 20 runs under normalization and logarithmic transformation, respectively.

The forecasting models of TOVAFAF for China’s various provincial-level administrative regions were established employing BP, ELM, PSO-BP, PSO-ELM, EPSO-BP, and EPSO-ELM algorithms under their respective parameter settings. To evaluate and dissect the modeling efficacy, R^2^ served as a metric to assess the goodness of fit for the models on the training set, whereas MRE was utilized to ascertain the reliability of the models’ forecasting capabilities on the validation set. Given that the initial weights of the neural networks are randomly assigned and vary from run to run, the aforementioned models were executed 20 times, and the mean value of the experimental results was calculated to assess the overall performance of various models. Additionally, the standard deviation (STD) of the results from these 20 runs was leveraged to evaluate the stability of the various models. To fully evaluate and analyze the modeling efficacy of various models, the above six neural network algorithms were divided into Group 1 (BP, PSO-BP, and EPSO-BP) and Group 2 (ELM, PSO-ELM, and EPSO-ELM), and three sets of comparative experiments were carried out. It should be noted that the modeling methods of Group 1 belong to the BP neural network series, while the modeling methods of Group 2 belong to the ELM series. EPSO and PSO are two different optimization mechanisms. Normalization and logarithmic transformation are two different data transformations, which only apply to the NTL indices. The specific content and purpose of comparative experiments are shown in Fig. 3. Table 3 lists the modeling results of various models established based on normalized and logarithmic transformed multiple NTL indices. Figure 4 and Fig. 5 visualize the modeling results, thereby making the results more intuitive.

**Table 3:** Modeling results (mean ± STD) of various models within 20 runs.

Model		BP	ELM	PSO-BP	PSO-ELM	EPSO-BP	EPSO-ELM
Normalization	R^2^	0.9053 ± 0.0318	0.8139 ± 0.0155	0.9182 ± 0.0164	0.8306 ± 0.0175	0.9383 ± 0.0038	0.8494 ± 0.0038
	MRE (%)	35.28 ± 7.75	44.29 ± 8.97	20.67 ± 1.17	22.96 ± 1.40	15.53 ± 0.55	21.09 ± 0.67
Logarithmic transformation	R^2^	0.9100 ± 0.0247	0.8809 ± 0.0097	0.9276 ± 0.0225	0.8854 ± 0.0060	0.9522 ± 0.0049	0.8936 ± 0.0013
	MRE (%)	33.94 ± 5.41	37.90 ± 8.16	20.21 ± 0.94	20.76 ± 0.80	15.18 ± 0.58	18.84 ± 0.30

#### Comparison and analysis of modeling methods within the group

In this section, the modeling methods within each group, i.e., Group 1 (BP, PSO-BP, and EPSO-BP) and Group 2 (ELM, PSO-ELM, and EPSO-ELM), were compared and analyzed to elucidate the modeling efficacy of the same neural network under different optimization mechanisms.

Regarding the modeling methods of Group 1, under both normalization and logarithmic transformation across the 20 runs, Fig. 4(a) and (b) illustrate that the R^2^ values of EPSO-BP exceed those of PSO-BP and BP in the majority of cases. Additionally, the R^2^ values of PSO-BP are higher than those of BP in most instances. Figure 5(a) and (b) reveal that the MRE values of EPSO-BP are lower than those of PSO-BP and BP in every case of the 20 runs, and the MRE values of PSO-BP are significantly lower than those of BP across all 20 runs. Furthermore, under the 20 runs, the fluctuation amplitudes of the R^2^ and MRE curves of EPSO-BP are smaller than those of PSO-BP and BP, and the fluctuation amplitudes of the R^2^ and MRE curves of PSO-BP are smaller than those of BP. Table 3, Fig. 4(c), and Fig. 5(c) demonstrate that the STD of R^2^ and MRE for EPSO-BP over the 20 runs is the lowest among the modeling methods of Group 1, and the STD of R^2^ and MRE for PSO-BP is less than that of BP, indicating that EPSO-BP possesses the best stability, and the stability of PSO-BP is superior to that of BP. These results confirm that EPSO-BP achieves the best modeling efficacy, and the modeling efficacy of PSO-BP surpasses that of BP.

It can be seen from Table 3, Fig. 4, and Fig. 5 that the modeling efficacy of the methods in Group 2 is the same as that of the methods in Group 1. Specifically, the modeling efficacy of EPSO-ELM is the best among the methods in Group 2, and the modeling efficacy of PSO-ELM is superior to that of ELM.

#### Comparison and analysis of modeling methods between the groups

This section compared and analyzed the corresponding modeling methods between the Group 1 (BP, PSO-BP, and EPSO-BP) and Group 2 (ELM, PSO-ELM, and EPSO-ELM), so as to elucidate the modeling efficacy of different neural networks under the same optimization mechanism.

Figure 4(a) shows that under normalization, the R^2^ values of EPSO-BP, PSO-BP, and BP are separately higher than those of EPSO-ELM, PSO-ELM, and ELM across all 20 runs. Figure 4(b) shows that under logarithmic transformation, the R^2^ values of EPSO-BP are significantly higher than those of EPSO-ELM in every case of the 20 runs, and the R^2^ values of PSO-BP, and BP are separately higher than those of PSO-ELM and ELM in most cases. The above results indicate that the fitting goodness of the BP neural network series models is superior to that of the ELM series models. Under both normalization and logarithmic transformation within the 20 runs, the MRE values of EPSO-BP are significantly lower than those of EPSO-ELM in every case, and the MRE values of PSO-BP and BP are separately lower than those of PSO-ELM and ELM in every case in the majority of cases, as shown in Fig. 5(a) and (b). The above results indicate that the forecasting capabilities of the BP neural network series models are better than those of the ELM series models. As can be seen from Table 3, Fig. 4, and Fig. 5, the fluctuation amplitudes and STD of the R^2^ and MRE values for the modeling methods in Group 1 are not significantly different from those of the corresponding methods in Group 2, which indicates that the stability of BP and ELM series models is similar. Based on the above results, it can be concluded that the modeling efficacy of the BP neural network series models surpasses that of the ELM series models.

#### Comparison and analysis of modeling methods under different data transformations

This section compared and analyzed the modeling results of various methods under normalization and logarithmic transformation to elucidate the modeling efficacy of neural networks under different data transformations.

As shown in Table 3 and Fig. 4(c), across the 20 runs, the mean values of R^2^ for various methods under logarithmic transformation are higher than those of corresponding methods under normalization, which indicates that the fitting goodness of the neural networks under logarithmic transformation is better than that under normalization. It can be seen from Table 3 and Fig. 5(c) that the mean values of MRE for various methods under logarithmic transformation within the 20 runs are lower than those of corresponding methods under normalization, which indicates that forecasting capabilities of the neural network models under logarithmic transformation are superior to those under normalization. Additionally, Table 3, Fig. 4(c), and Fig. 5(c) show that the STD values of the R^2^ and MRE for BP, ELM, PSO-ELM, and EPSO-ELM under logarithmic transformation over the 20 runs are lower than those under normalization, and the STD values of the MRE for PSO-BP under logarithmic transformation are lower than those under normalization, which indicates that the stability of neural network models under logarithmic transformation is better than that under normalization. Therefore, the above results demonstrate that the modeling efficacy of neural networks under logarithmic transformation surpasses that under normalization.

### Evaluation and analysis of forecasting performance

The conclusions drawn from the above modeling results are as follows: (1) Neural networks optimized by the EPSO mechanism exhibit better modeling efficacy than those optimized by the PSO mechanism; (2) The BP neural network series models demonstrate superior modeling efficacy compared to the ELM series models; (3) Neural networks under logarithmic transformation achieve better modeling efficacy than those under normalization. To further validate the above conclusions, four neural network models with better modeling efficacy, i.e., PSO-BP, PSO-ELM, EPSO-BP, and EPSO-ELM, were selected to forecasting the TOVAFAF for China’s various provincial-level administrative regions in 2023. During the forecasting process, each neural network model was run 20 times to mitigate the impact of random errors on the results. The forecasting performance of various models was evaluated and analyzed using RE, MRE, STD, and the R^2^ for the linear relationship between the forecasting values and the actual values. It is important to highlight that the study’s scope does not encompass five provincial-level administrative regions of China, namely Beijing, Shanghai, Hong Kong, Macao, and Taiwan. The detailed explanations for this exclusion are provided in Sec. Study Area and Data Sources.

#### Forecasting performance for each provincial-level administrative region in 2023

**Fig. 6:**
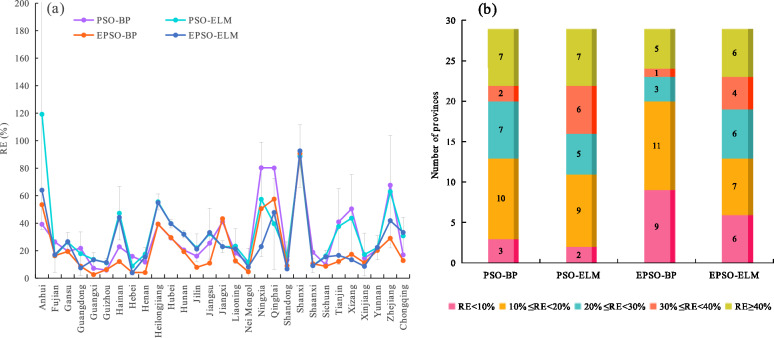
Forecasting results of various models under normalization for each provincial-level administrative region in 2023: (**a**) the relative error (RE) of various models for each provincial-level administrative region; (**b**) the number of provincial-level administrative regions at various levels of RE.

**Fig. 7:**
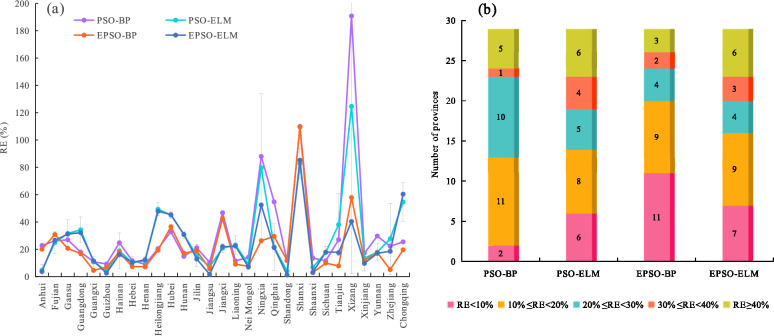
Forecasting results of various models under logarithmic transformation for each provincial-level administrative region in 2023: (**a**) the relative error (RE) of various models for each provincial-level administrative region; (**b**) the number of provincial-level administrative regions at various levels of RE.

The RE was utilized to evaluate the forecasting performance of various models on TOVAFAF for each provincial-level administrative region. Figure 6(a) and Fig. 7(a) show the RE of various models under normalization and logarithmic transformation for each provincial-level administrative region in 2023, respectively, in which the RE values are the mean of the RE values obtained by running each model 20 times. Moreover, Fig. 6(b) and Fig. 7(b) show the number of provincial-level administrative regions at various levels of RE under normalization and logarithmic transformation, respectively.

Upon reviewing Fig. 6(b) and Fig. 7(b), it can be seen that under normalization and logarithmic transformation, there are 23 and 24 provincial-level administrative regions with RE values below 30% for the EPSO-BP model, respectively, exceeding the performance of other models. Specifically, there are 9 and 11 provincial-level administrative regions with RE values below 10% for the EPSO-BP model under normalization and logarithmic transformation, respectively, also surpassing other models under the same conditions. These outcomes imply that the EPSO-BP model provides the best forecasting performance. For the EPSO-ELM model, 19 and 20 provincial-level administrative regions displayed RE values below 30% under normalization and logarithmic transformation, respectively. For the PSO-ELM model, 16 and 19 provincial-level administrative regions showed RE values below 30% under normalization and logarithmic transformation, respectively, less than those of the EPSO-ELM model. The above results suggest that neural networks optimized using the EPSO mechanism provide superior forecasting performance compared to those optimized by the PSO mechanism. For the PSO-BP model, there are 20 and 23 provincial-level administrative regions with RE values below 30% under normalization and logarithmic transformation, respectively, more than those of the PSO-ELM model. The results indicate that under the same optimization mechanism, the BP neural network series models perform better than the ELM series models in forecasting the TOVAFAF for China’s various provincial-level administrative regions. Moreover, for PSO-BP, PSO-ELM, EPSO-BP, and EPSO-ELM, the number of provinces with RE below 30% under logarithmic transformation are more than those under normalization, respectively. Specifically, there are 6, 11, and 7 provincial-level administrative regions with RE values below 10% for PSO-ELM, EPSO-BP, and EPSO-ELM under logarithmic transformation, respectively, also more than those under normalization. The results demonstrate that the neural network models under logarithmic transformation exhibit better forecasting performance than those under normalization.

#### Overall forecasting performance for various provincial-level administrative regions in 2023

The overall forecasting performance of various models was evaluated using MRE, STD, and the R^2^ for the linear relationship between the forecasting values and the actual values. It should be noted that MRE values are the mean of MRE obtained by running each model 20 times, and R^2^ is the determination coefficient for the linear relationship between all actual values and forecasting values obtained by running each model 20 times.

**Fig. 8:**
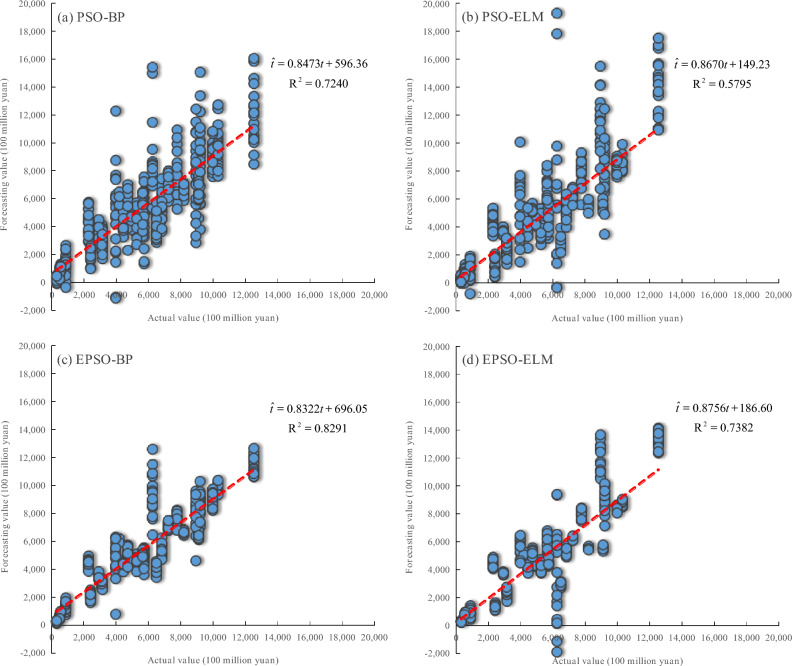
The linear relationship between the actual values and the forecasting values of various models under normalization in 2023: (**a**) PSO-BP, (**b**) PSO-ELM, (**c**) EPSO-BP, and (**d**) EPSO-ELM.

**Fig. 9:**
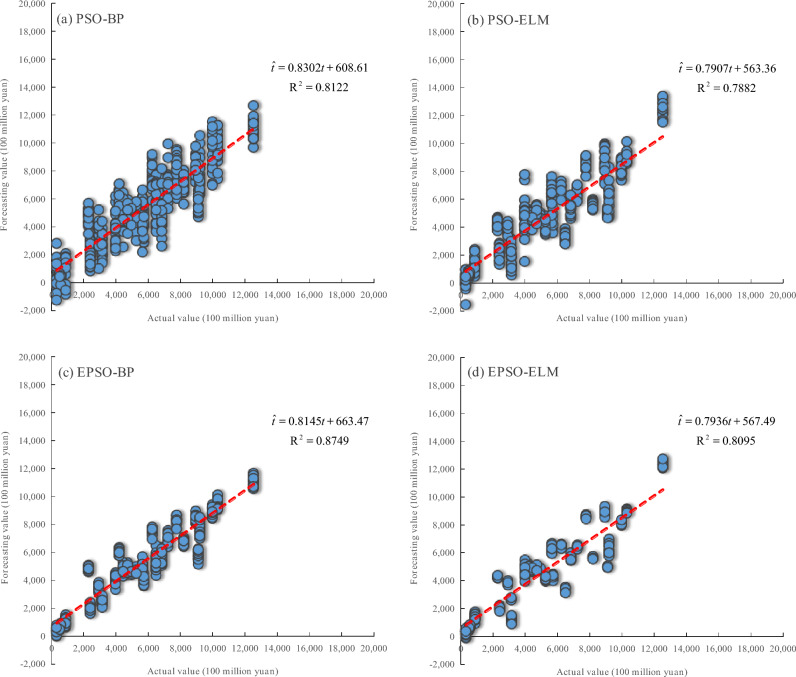
The linear relationship between the actual values and the forecasting values of various models under logarithmic transformation in 2023: (**a**) PSO-BP, (**b**) PSO-ELM, (**c**) EPSO-BP, and (**d**) EPSO-ELM.

As shown in Table 4, under normalization and logarithmic transformation, the MRE and STD values of EPSO-BP and EPSO-ELM are significantly lower than those of PSO-BP and PSO-ELM, respectively. And Fig. 8 and Fig. 9 show that the R^2^ values for the linear relationship between the actual values and forecasting values obtained by EPSO-BP and EPSO-ELM are higher than those of PSO-BP and PSO-ELM, respectively. The results further indicate that the neural network models optimized by the EPSO mechanism exhibit better forecasting performance than those optimized by the PSO mechanism.

**Table 4:** Overall forecasting performance (mean ± STD) of various models in 2023.

Model	PSO-BP	PSO-ELM	EPSO-BP	EPSO-ELM
MRE (%) under normalization	30.12 ± 3.46	33.07 ± 4.95	21.55 ± 1.44	26.49 ± 1.37
MRE (%) under logarithmic transformation	32.19 ± 7.99	29.03 ± 5.16	20.65 ± 1.76	23.50 ± 1.56

It can be seen from Table 4 that the MRE values of EPSO-BP are both lower than those of EPSO-ELM under normalization and logarithmic transformation, and the MRE value of PSO-BP is lower than that of PSO-ELM under normalization. In addition, Fig. 8 and Fig. 9 show that under normalization and logarithmic transformation, the R^2^ values for the linear relationship between the actual values and forecasting values obtained by EPSO-BP and PSO-BP are higher than those of EPSO-ELM and PSO-ELM, respectively. On the whole, the results further demonstrate that the BP neural network series models provide superior forecasting performance on the TOVAFAF for China’s various provincial-level administrative regions compared to the ELM series models.

As can be seen from Table 4, the MRE value of the PSO-BP model under logarithmic transformation is higher than its MRE value under normalization, but the MRE values of PSO-ELM, EPSO-BP, and EPSO-ELM under logarithmic transformation are all lower than their MRE values under normalization. Although the STD values of PSO-ELM, EPSO-BP, and EPSO-ELM under logarithmic transformation are slightly higher than their STD values under normalization, the difference between the two is not significant, indicating that the stability of PSO-ELM, EPSO-BP, and EPSO-ELM under logarithmic transformation is basically consistent with the stability under normalization. Moreover, Fig. 8 and Fig. 9 show that the R^2^ values for the linear relationship between the actual values and forecasting values obtained by PSO-BP, PSO-ELM, EPSO-BP, and EPSO-ELM under logarithmic transformation are all higher than those under normalization. As a whole, the results further evidence that the neural network models under logarithmic transformation achieve better forecasting performance than those under normalization.

In our previous research^36^, the PSO-ELM model, established on the basis of four NTL indices (TNLI, LPQI, LPRI, and NLSDSI) under normalization, obtains the MRE with 32.20% and the R^2^ with 0.6460 for the linear relationship between the actual and forecasting values when forecasting the TOVAFAF in various provinces of China in 2023. In this research, the MRE values of PSO-BP, EPSO-BP, and EPSO-ELM established based on all constructed eight NTL indices under normalization are all lower than that of the PSO-ELM model in our previous research, as shown in Table 4. And the the R^2^ values for the linear relationship between the actual and forecasting values obtained by PSO-BP, EPSO-BP, and EPSO-ELM under normalization in this research are all higher that of PSO-ELM in our previous research, as shown in Fig. 8. These results also further indicate that BP neural network and EPSO mechanism are more capable of exploring the potential nonlinear relationship between NTL data and the TOVAFAF than ELM and PSO mechanism. Furthermore, about the MRE and the R^2^ for the linear relationship between the actual and forecasting values, Table 4 and Fig. 9 show that PSO-BP, PSO-ELM, EPSO-BP, and EPSO-ELM under logarithmic transformation in this research all perform better than the PSO-ELM model under normalization in our previous research. This also further proves that logarithmic transformation is more effective than normalization in enhancing the relationship between NTL data and the TOVAFAF, thereby improving the forecasting performance of the models for the TOVAFAF. As evidenced by the forecasting results for TOVAFAF across various provinces of China in 2023, the EPSO-BP model under logarithmic transformation exhibits the highest forecasting accuracy, with the MRE of 20.65% and the R^2^ value of 0.8749 for the linear relationship between the actual and forecasting values. Compared with the PSO-ELM model in our previous research^36^, the MRE of the EPSO-BP model in this research decreases by 11.55 percentage points, and the R^2^ for the linear relationship between the actual and forecasting values increased by 35.38%, which indicates that the forecasting performance of EPSO-BP on the TOVAFAF in various provinces of China has been significantly improved.

Furthermore, it can be seen from Fig. 8 and Fig. 9 that there are some negative values among the models’ forecasting values for the TOVAFAF, which is not in line with objective fact in practice. According to statistical analysis, these negative values mainly appear in individual provinces with low TOVAFAF values, such as Xizang and Ningxia. The core of the above problem stems from the extrapolation errors of neural network models caused by uneven sample distribution. The specific reason is that the annual TOVAFAF value of most provinces reaches hundreds of billions of yuan, while the sample proportion of low-value provinces with annual TOVAFAF values in the tens of billions of yuan, such as Xizang and Ningxia, is relatively small, which leads to the model’s insufficient fitting to the sparse and low-value samples. Such data bias may cause the model to generate unreasonable negative values when forecasting low-value targets. From the perspective of forecasting models, PSO-BP and PSO-ELM models generate relatively more negative values during the forecasting process compared to the EPSO-BP and EPSO-ELM models. Especially, it can be seen from Fig. 8(c) and Fig. 9(c) that the EPSO-BP model produces almost no negative forecasting values. This further demonstrates that the EPSO mechanism proposed in this paper can significantly improve the forecasting performance of single-hidden-layer neural network models for TOVAFAF.

## Conclusions

To further explore the potential nonlinear relationship between NTL data and the TOVAFAF, building upon our previous research^36^, the eight originally constructed NTL indices (TNLI, ANLI, LPQI, LPRI, ALPLI, NLSDI, NLVI, and NLSDSI) processed by normalization and logarithmic transformation were taken as multiple input variables in this research, respectively. And a series of forecasting models for the TOVAFAF were constructed using neural networks and optimization mechanisms. The conclusions of this study are as follows:

- The experimental results further verify that the neural network algorithms can effectively characterize the potential nonlinear relationship between NTL data and the TOVAFAF, which is consistent with our previous research conclusions;
- Neural networks optimized using the EPSO mechanism exhibit superior forecasting performance compared to those optimized by the PSO mechanism; the BP neural network series models demonstrate better forecasting performance than the ELM series models; and neural networks under logarithmic transformation achieve improved forecasting performance over those under normalization;
- The EPSO-BP model under logarithmic transformation exhibits the best forecasting performance on the TOVAFAF of various provinces of China in 2023, with the MRE of 20.65% and the R^2^ value of 0.8749 for the linear relationship between the actual and forecasting values.

The EPSO mechanism proposed in this paper significantly enhances the forecasting accuracy of single-hidden-layer neural network models for TOVAFAF in various provinces of China, specifically the EPSO-BP model. However, verification has been confined to the provincial level and has not been extended to finer spatial resolutions (e.g., prefectural or county levels), which may limit its applicability to localized agricultural planning. Furthermore, reliance on NPP-VIIRS NTL data, whose time series begins in 2012, may limit the robustness of the model for long-term trend analysis. In future work, studies will be conducted more deeply at the county or prefectural level. Moreover, through cross-sensor calibration, DMSP-OLS NTL data (1992–2013) will be integrated with NPP-VIIRS NTL data to extend the temporal coverage, increase sample size, and thereby enhance model robustness for long-term trend analysis.

## Data Availability

The data and codes in this study can be requested from the first author or corresponding author via email upon reasonable request.
